# Correspondence of categorical and feature‐based representations of music in the human brain

**DOI:** 10.1002/brb3.1936

**Published:** 2020-11-08

**Authors:** Tomoya Nakai, Naoko Koide‐Majima, Shinji Nishimoto

**Affiliations:** ^1^ Center for Information and Neural Networks National Institute of Information and Communications Technology Suita Japan; ^2^ Graduate School of Frontier Biosciences Osaka University Suita Japan; ^3^ AI Science Research and Development Promotion Center National Institute of Information and Communications Technology Suita Japan; ^4^ Graduate School of Medicine Osaka University Suita Japan

**Keywords:** fMRI, MTF model, music genre, STG

## Abstract

**Introduction:**

Humans tend to categorize auditory stimuli into discrete classes, such as animal species, language, musical instrument, and music genre. Of these, music genre is a frequently used dimension of human music preference and is determined based on the categorization of complex auditory stimuli. Neuroimaging studies have reported that the superior temporal gyrus (STG) is involved in response to general music‐related features. However, there is considerable uncertainty over how discrete music categories are represented in the brain and which acoustic features are more suited for explaining such representations.

**Methods:**

We used a total of 540 music clips to examine comprehensive cortical representations and the functional organization of music genre categories. For this purpose, we applied a voxel‐wise modeling approach to music‐evoked brain activity measured using functional magnetic resonance imaging. In addition, we introduced a novel technique for feature‐brain similarity analysis and assessed how discrete music categories are represented based on the cortical response pattern to acoustic features.

**Results:**

Our findings indicated distinct cortical organizations for different music genres in the bilateral STG, and they revealed representational relationships between different music genres. On comparing different acoustic feature models, we found that these representations of music genres could be explained largely by a biologically plausible spectro‐temporal modulation‐transfer function model.

**Conclusion:**

Our findings have elucidated the quantitative representation of music genres in the human cortex, indicating the possibility of modeling this categorization of complex auditory stimuli based on brain activity.

## Graphical abstract

Discrete music categories were represented in the bilateral superior temporal gyri. A feature‐brain similarity analysis quantitatively showed that such representation was explained by the spectro‐temporal modulation‐transfer function model.

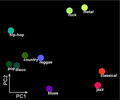

## 1 INTRODUCTION

Humans tend to categorize auditory stimuli into discrete classes. Such class labels encompass animal species, language, musical instrument, and music genre. Of these, music genre is a common class label for understanding how humans recognize and categorize music, and it is widely used in studies of music information retrieval (Sturm, 2012). However, there remains considerable uncertainty as to how such genre categories are perceived from complex auditory stimuli and how the human brain subserves this categorization. Neuroimaging studies have decoded music genres from brain activity using support vector machines (SVM) (Case y, 2017; Ghaemmaghami & Sebe, 2016; Sengupta et al., 2018); however, these studies did not clarify how cortical representations of music genres contribute to genre classification.

Previous studies have examined the representations of general music‐related features, for example, loudness, in the brain (Alluri et al., 2012; Hoefle et al., 2018; Toiviainen et al., 2014). Alluri et al. (2012) reported significant correlation between activation in the bilateral superior temporal gyrus (STG) with features of timbre, harmony, and rhythm. Moreover, Toiviainen et al. (2014) revealed involvement of the bilateral STG in the decoding of timbral features. In contrast, cochlear and spectro‐temporal modulation‐transfer function (MTF) models have been widely used as biologically plausible models for the acoustic representation of STG (de Heer et al., 2017; Norman‐Haignere et al., 2015; Patil et al., 2012; Santoro et al., 2014, 2017). The cochlear model represents tonotopic information received through auditory pathways (de Heer et al., 2017; Saenz & Langers, 2014), but modulation‐selective responses have been detected in the primary auditory cortex in ferrets (Depireux et al., 2001) and humans (Hullett et al., 2016; Langers et al., 2003; Pasley et al., 2012; Schonwiesner & Zatorre, 2009). Moreover, the MTF model has been applied to explain brain activation differences between 2‐s excerpts of music and voices in STG (Norman‐Haignere et al., 2015) and between simple tones of various musical instruments (Patil et al., 2012). However, it is unclear whether these biologically plausible models can explain significant variance in the brain activity patterns of genre categories comprising complex auditory stimuli. Further, the process by which various music genre categories are organized in a fine‐scale manner is not well understood.

Recent neuroimaging studies have employed voxel‐wise encoding/decoding models (Naselaris et al., 2011) to investigate sensory and higher‐order cortical representations, including visual (Kay et al., 2008; Nishimoto et al., 2011) and auditory modalities (Allen et al., 2018; de Heer et al., 2017; Huth et al., 2016). One advantage of an encoding/decoding model approach is its ability to use the same dataset to compare the performances of several competing theoretical models. de Heer et al. (2017) modeled brain activity during passive story listening and conducted encoding model fitting using cochlear, phoneme, and semantic features. Allen et al. (2018) conducted encoding model fitting with multiple acoustic features and reported the advantage of a timbre model for predicting auditory cortex activity induced by simple instrumental tones. Such approaches can be employed to further assess whether a biologically plausible model is more effective in predicting brain activation underlying categorical representation.

**Figure 1:**
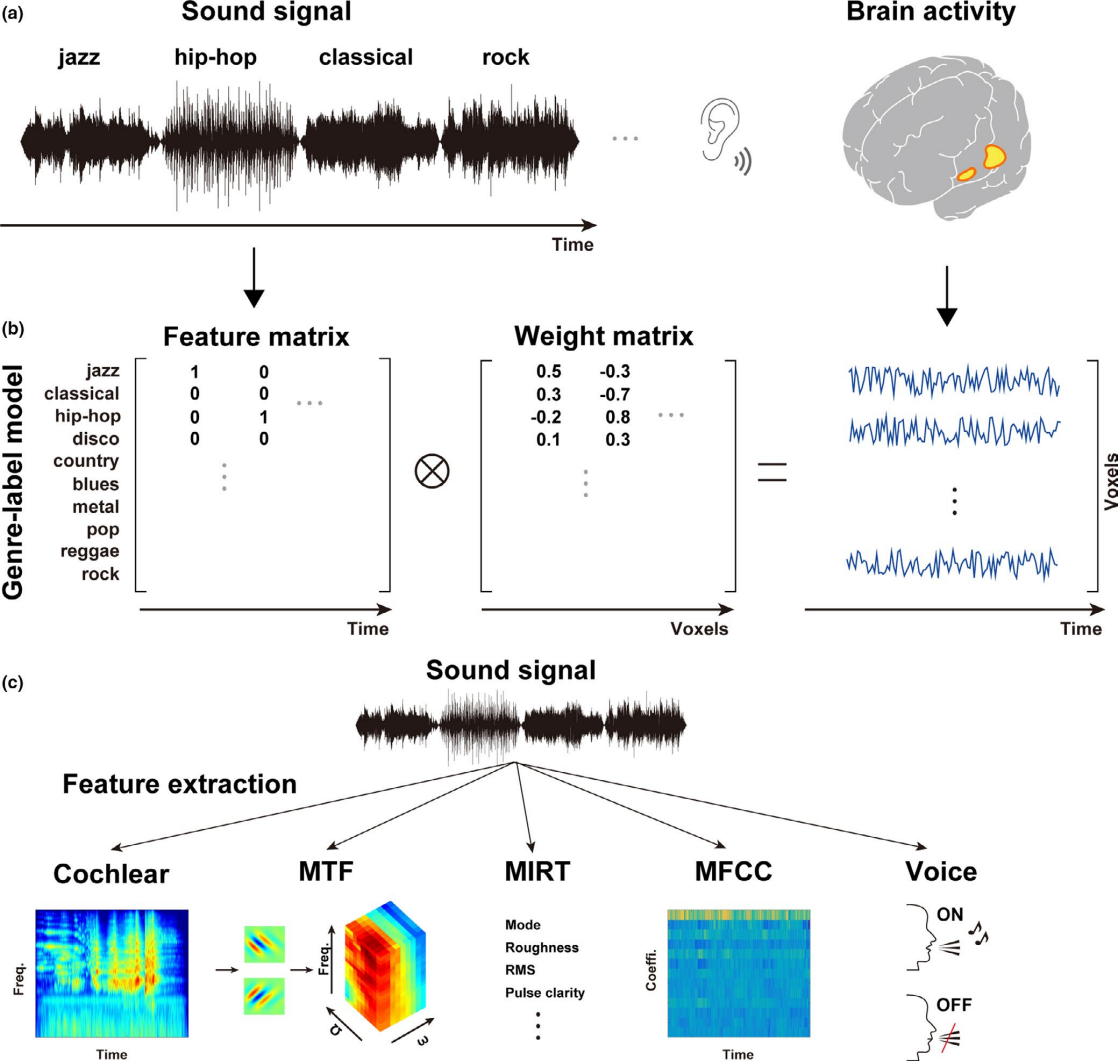
Schematic image showing the research paradigm of the present study. (**A)**Participants listened passively to the naturalistic music stimuli of 10 music genres, and evoked brain activity was measured using fMRI.**(B)**Voxel‐wise brain activity was modeled as a feature matrix (music genre labels) times a weight matrix. Regularized linear regression was used to estimate optimal weights.**(C)**Five different acoustic models were used to explain genre‐specific brain activation patterns. Cochlear, modulation‐transfer function (MTF), MIRtoolbox (MIRT), mel‐frequency cepstrum coefficients (MFCC), and voice features were extracted from the original sound signals. Each model is described in the Methods section.

Consequently, we used an encoding and decoding model approach to examine brain activity induced by music stimuli from different genre categories and examined the detailed cortical organization underlying each genre representation and how acoustic features can explain such categorical organization. Accordingly, five participants listened passively to naturalistic music stimuli representing 10 different music genres, and evoked brain activity was measured using fMRI (Figure 1 A). We hypothesized that music pieces are represented in a genre‐specific way in the human brain and that such categorical representation reflects how the cortical response pattern to acoustic features matches the acoustic property of individual music genre categories. We examined specific cortical activation patterns based on predefined genre labels (Figure 1B) and showed how different genre categories are organized on the cortical surface. We then extracted acoustic features using two biologically plausible models (cochlear, MTF), two music‐related models [MIRtoolbox (MIRT) and mel‐frequency cepstral coefficient (MFCC)], and one voice‐related model (voice model) (Figure 1C). MIRT features have been used to describe music‐induced activation patterns in the bilateral STG (Alluri et al., 2012; Toiviainen et al., 2014). MFCC features were developed and are predominantly used for speech recognition (Güçlü et al., 2016). Since distinct activity patterns were reported for the categorical perception of the human voice and musical instruments (Leaver & Rauschecker, 2010), we used the voice model to test whether genre‐related brain activity can be explained merely by the effect of voice stimuli.

To investigate which acoustic features most accurately explain the categorical organization in the brain, we developed a novel technique of calculating the similarity of feature‐based and brain‐based representation [feature–brain similarity (FBS)]. FBS assesses how cortical voxels realize the categorical representation of individual music genres through target acoustic features by measuring similarities between the cortical weight vector of corresponding acoustic features and the reference acoustic feature vector of each music genre. FBS can be calculated in each voxel and is distinct from representational similarity analysis (Kriegeskorte et al., 2008) that calculates similarity across different categories based on multi‐voxel patterns. Therefore, FBS is suitable for examining categorical representation in the voxel‐wise encoding modeling employed in our study. We further investigated the representational specificity between music genres by performing genre classification with the brain activity, behavior, and extracted features. Finally, we tested whether these acoustic features could capture such representational differences of music genres. Preliminary findings have been published in the IEEE International Conference on Systems, Man, and Cybernetics (IEEE SMC 2018) (Nakai et al., 2018).

## 2 MATERIALS AND METHODS

### 2.1 Participants

Five healthy participants (referred to as ID01‐05; age range 23–33; 2 females; music experience, 4–15 years) with normal hearing participated in the MRI and behavioral experiments. An additional 21 participants (age 20–24; 5 females; music experience, 0–15 years) participated only in the behavioral experiment. A questionnaire was used to assess the number of years that participants trained on their primary instrument; this was used as the index of musical experience. Informed consent was obtained from all participants prior to their participation. This experiment was approved by the ethics and safety committee of the National Institute of Information and Communications Technology in Osaka, Japan.

### 2.2 Stimuli and task

Music stimuli from 10 genres (blues, classical, country, disco, hip‐hop, jazz, metal, pop, reggae, and rock) were taken at random from the GTZAN music genre dataset (http://marsyasweb.appspot.com/download/data_sets/) (Tzanetakis & Cook, 2002). A total of 54 music pieces (30 s, 22,050 Hz) were selected from each genre, providing 540 music pieces. A 15‐s music clip was selected at random from each music piece. For each clip, 2 s of fade‐in and fade‐out effects were applied, and the overall signal intensity was normalized in terms of the root mean square (RMS).

Each experiment consisted of 18 runs: 12 were considered as training runs, and 6 were considered as test runs. Each run consisted of 40 music clips and lasted 10 min in total. At the beginning of each run, 15 s of dummy scanning was acquired; this was omitted from each analysis. A total of 480 of the music clips were used in the training runs, and the remaining 60 were reserved for the test runs. For the purposes of data reproducibility, a set of 10 music clips was presented four times in the same order as part of each test run. There was no repetition in the training runs. The clip order was randomized across the experiment. During scanning, participants were asked to fixate on a fixation cross at the center of the screen and to listen to the music clips through MRI‐compatible insert earphones (Model S14, Sensimetrics). This model can attenuate scanner noise and has been widely used in previous MRI studies with auditory stimuli (Allen et al., 2018; de Heer et al., 2017; Huth et al., 2016; Kell et al., 2018; Norman‐Haignere et al., 2015; Santoro et al., 2017). After each 10 min run, we asked the participants to describe their physical condition, and we allowed a 1–2 min break if they felt fatigue or sleepiness. After all runs on each day, we asked the participants whether they fell asleep during scanning. According to their self‐reports, nobody slept during the experiments. The experiment was executed over the course of three days, with six runs performed each day.

### 2.3 MRI data acquisition

Scanning was performed using a 3.0 T MRI scanner (TIM Trio; Siemens, Erlangen, Germany) equipped with a 32‐channel head coil. For functional scanning, we scanned 68 interleaved axial slices with a thickness of 2.0 mm without a gap using a T2*‐weighted gradient echo multi‐band echo‐planar imaging (MB‐EPI) sequence (Moeller et al., 2010) (repetition time (TR) = 1,500 ms, echo time (TE) = 30 ms, flip angle (FA) = 62°, field of view (FOV) = 192 × 192 mm^2^, voxel size = 2 × 2 × 2 mm^3^, multi‐band factor = 4). A total of 410 volumes were obtained for each run. For anatomical reference, we acquired high‐resolution T1‐weighted images of the whole brain from all participants using a magnetization prepared rapid acquisition gradient echo sequence (MPRAGE, TR = 2,530 ms, TE = 3.26 ms, FA = 9°, FOV = 256 × 256 mm^2^, voxel size = 1 × 1 × 1 mm^3^).

### 2.4 Feature assignment

To examine the representational basis of music, the following features were assigned to the stimulus sounds (Figure 1).

#### 2.4.1 Genre‐label features

The genre‐label features consisted of 10 features corresponding to the 10 music genres. Values of either 1 or 0 were assigned to the entire time duration of a 15 s music clip (consisting of 10 TRs) to denote the music genre of the target music clip.

#### 2.4.2 Cochlear and MTF features

A sound cochleogram was generated by processing the stimulus sounds using a bank of 128 overlapping band‐pass filters, spanning from 100 to 8,000 Hz (Ellis, 2009). The window size was set to 25 ms, with the hop size set to 10 ms. The filter output averaged across 1.5 s (TR) was used as a feature in the cochlear model.

Then, we extracted MTF features as performed by Chi et al. (2005). For each cochleogram, a convolution with modulation‐selective filters was calculated. The outputs of the two filters with orthogonal phases (quadrature pairs) were squared and summed to yield the local modulation energy (Nishimoto et al., 2011). The local modulation energy was log‐transformed, averaged across 1.5 s, and further averaged within each of the 20 nonoverlapping frequency ranges logarithmically spaced in the frequency axis. The filter outputs of the upward and downward sweep directions were then averaged. Modulation‐selective filters were tuned to 10 spectral modulation scales [Ω = (0.35, 0.50, 0.71, 1.0, 1.41, 2.0, 2.83, 4.0, 5.66, 8.0) cyc/oct] and 10 temporal modulation rates [ω = (2.8, 4.0, 5.7, 8.0, 11.3, 16.0, 22.6, 32.0, 45.3, 64.0) Hz]. To reduce the computational burden, the resultant 20 × 10 × 10 = 2000 features were reduced to 302 features using principal component analysis (PCA), which preserved 99% of the variance of the original features. The number of features used in the Cochlear and MTF models was 128 and 302, respectively.

#### 2.4.3 MIRT and MFCC features

For the MIRT model, the MIR toolbox was used to extract multiple music‐related features from the dataset (Lartillot et al., 2008). Consistent with a previous neuroimaging study on music (Alluri et al., 2012), we extracted the following 24 features: RMS energy as the loudness feature; zero‐crossing rate, high energy–low energy ratio, spectral centroid, spectral roll‐off, spectral entropy, spectral flatness, roughness, spectral spread, spectral flux, and sub‐band flux (with nine sub‐bands) as timbral features; pulse clarity, fluctuation centroid, and fluctuation entropy as rhythm features; and mode and key clarity as tonal features. For loudness and timbral features, the frame duration was set to 25 ms, with a 50% overlap between the two adjacent frames. For rhythm and tonal features, the frame duration was set to 3 s, with a 33% overlap. Each feature was averaged across 1.5 s. In addition, we used the MIR toolbox to extract MFCC features with 12 channels (Lartillot et al., 2008). Feature extraction of the MIRT and MFCC models was also restricted within 100–8,000 Hz. The number of features used in the MIRT and MFCC models was 24 and 12, respectively.

#### 2.4.4 Voice features

The voice features consisted of two features corresponding to whether each music piece contained voice stimuli. Values of either 1 or 0 were assigned to the entire time duration of a 15 s music clip (consisting of 10 TRs) to denote the presence or absence of voice.

### 2.5 Data analyses

#### 2.5.1 fMRI data preprocessing

Motion correction was performed for each run using the Statistical Parametric Mapping toolbox (SPM8; Wellcome Trust Centre for Neuroimaging, London, UK; http://www.fil.ion.ucl.ac.uk/spm/). All volumes were aligned to the first EPI image for each participant. Low‐frequency drift was removed using a median filter with a 240‐s window. To augment model fitting accuracy, the response for each voxel was normalized by subtracting the mean response and then scaling it to the unit variance. We used FreeSurfer (Dale et al., 1999; Fischl et al., 1999) to identify the cortical surfaces from the anatomical data and register them with the voxels of the functional data. We used only cortical voxels as targets of the analysis for each participant. For each participant, we used the voxels identified in the cerebral cortex in the analysis (53,421–64,700 voxels per participant).

#### 2.5.2 Voxel‐wise encoding model fitting

For each of the above models, cortical activation for each voxel was fitted using a set of linear temporal filters that captured the slow hemodynamic response and its coupling with brain activity (Nishimoto et al., 2011). A feature matrix F_E_ [T × 5N] was modeled using concatenated sets of [T × N] feature matrices with five temporal delays of 1.5, 3, 4.5, 6, and 7.5 s (T, # of samples; N, # of features). The cortical response R_E_ [T × V] was modeled using the feature matrix F_E_ times the weight matrix W_E_ [5N × V] (V, # of voxels):

$${\hat{R}}_{E}=F_{E}W_{E}$$

We conducted an L2‐regularized linear regression using the training dataset (4,800 samples, 7,200 s) to obtain the weight matrix W_E_. The optimal regularization parameter was evaluated via random resampling of the training dataset into two subsets, with 80% of the dataset being used for model fitting and the remaining 20% being used for model validation. This random resampling procedure was repeated 10 times.

The test dataset comprised 600 samples (900 s). The signal‐to‐noise ratio was increased by averaging four repetitions of the test datasets. We calculated prediction accuracy by means of the Pearson's correlation coefficient between the predicted signal and the measured signal in the test dataset. The resulting *p* values were corrected for multiple comparisons within each participant using the false discovery rate (FDR) procedure (Benjamini & Hochberg, 1995). Mean prediction accuracy of each encoding model was calculated by averaging the prediction accuracy of all voxels within the participant‐specific region‐of‐interest mask (see below). The prediction accuracies of all models are summarized in Table 1. All model fitting and analyses were performed using custom software written on MATLAB. For data visualization on the cortical maps, pycortex was used (Gao et al., 2015).

**Table 1:** Prediction accuracy in each anatomical region

	Genre‐Label	Genre‐Label (with Voice regressor)	Cochlear	MTF	MIRT	MFCC	Voice
L. LSTG	0.079 ± 0.009	0.068 ± 0.017	0.070 ± 0.022	0.095 ± 0.017	0.074 ± 0.016	0.034 ± 0.007	0.078 ± 0.015
L. HG	0.153 ± 0.027	0.144 ± 0.023	0.159 ± 0.017	0.194 ± 0.040	0.159 ± 0.023	0.095 ± 0.027	0.098 ± 0.030
L. HS	0.222 ± 0.085	0.199 ± 0.076	0.200 ± 0.043	0.261 ± 0.050	0.223 ± 0.075	0.104 ± 0.038	0.169 ± 0.065
L. PT	0.121 ± 0.027	0.111 ± 0.028	0.104 ± 0.033	0.130 ± 0.027	0.114 ± 0.027	0.058 ± 0.017	0.114 ± 0.044
R. LSTG	0.100 ± 0.023	0.081 ± 0.026	0.102 ± 0.016	0.131 ± 0.029	0.100 ± 0.015	0.053 ± 0.009	0.103 ± 0.023
R. HG	0.152 ± 0.022	0.136 ± 0.034	0.163 ± 0.029	0.213 ± 0.045	0.161 ± 0.011	0.099 ± 0.018	0.125 ± 0.030
R. HS	0.198 ± 0.035	0.178 ± 0.026	0.188 ± 0.037	0.248 ± 0.040	0.205 ± 0.045	0.102 ± 0.048	0.145 ± 0.031
R. PT	0.116 ± 0.040	0.108 ± 0.041	0.131 ± 0.017	0.140 ± 0.069	0.118 ± 0.018	0.059 ± 0.023	0.090 ± 0.046

Note

Average prediction accuracies of six models across all participants (mean ± *SD*) calculated in the eight anatomical regions of interest. The prediction accuracy of the genre‐label model was also calculated by regressing out the voice effect. LSTG, lateral superior temporal gyrus; HG, Heschl's gyrus; HS, Heschl's sulcus; PT, planum temporale; MTF, modulation‐transfer function; MIRT, music information retrieval toolbox; MFCC, mel‐frequency cepstral coefficient.

#### 2.5.3 Genre‐representing region‐of‐interest (ROI) mask

To obtain robust estimates of the genre‐related cortical regions, we used the following resampling procedure: First, the training dataset was divided randomly into training samples (80%) and validation samples (20%). Using the optimal regularization parameter estimated in the analysis of the genre‐label model, we then performed encoding model fitting using the genre‐label features with the training samples and calculated the prediction accuracy with the validation samples. Model fitting was performed using L2‐regularized linear regression. This random resampling procedure was repeated 50 times, and the voxels showing significant prediction accuracy (FDR corrected) for more than 80% of the repetitions were selected for the ROI mask. We included 468 voxels in the ROI mask for participant ID01, 453 for participant ID02, 1,686 for participant ID03, 576 for participant ID04, and 530 for participant ID05. Unless otherwise indicated, the following analyses were all performed using the extracted ROI mask.

#### 2.5.4 Decoding of genre labels

In the decoding model, the cortical response matrix R_D_ [T × 5V] was obtained by concatenating the set of [T × V] response matrices with five temporal delays of 1.5, 3, 4.5, 6, and 7.5 s. The genre‐label matrix G [T × 10] was modeled using the cortical response matrix R_D_ times the weight matrix W_D_ [5V × 10]:

$$\hat{G}=R_{D}W_{D}$$

The weight matrix *W*_D_ was estimated using an L2‐regularized linear regression with the training dataset following the same procedure used for the encoding model fitting. We used linear regression rather than a categorical classifier, such as logistic regression, to maintain the similarity between the encoding and decoding analyses. To calculate the classification accuracy, we first assigned genre‐label indices (1 to 10) to each time point by taking the argmax of the decoded genre‐label matrix. Then, we estimated the representative genre‐label index for each music clip by the majority voting method (Dalwon et al., 2008). Specifically, the genre‐label that was most frequently assigned for all time points during a single music clip was regarded as a representative genre of that clip.

In the activity‐based approach, we obtained a response matrix *R*_D_ for each participant, whereas we used the feature matrix F_D_ [T × N] in the feature‐based approach.

#### 2.5.5 Behavioral experiment

To confirm that brain activation in response to music genres was related to the behavioral performance of genre classification, we performed additional behavioral experiments. These experiments were conducted in a soundproof room by the same participants who participated in the MRI experiments, as well as an additional 21 participants who had not taken part in the MRI experiments. Participants were first asked to listen to three original music clips (30 s) per genre as a reference; these clips were selected at random from the 460 clips not used in the MRI experiment. In this training session, correct music genres were informed to the participants. Participants then listened to the 60 music clips used as the test dataset in the MRI experiment and judged the music genre to which the target music clip belonged by filling in 1 of 10 cells on the answer sheet provided. Participants listened to each music clip only once and in the same order of presentation as in the fMRI experiment. Of the 21 non‐MRI participants, data from one participant were excluded because the average accuracy (30.0%) was outside the mean ± 3*SD range (and also outside the median 3*interquartile range) for all participants.

## 3 RESULTS

### 3.1 Genre‐representing cortical organization

**Figure 2:**
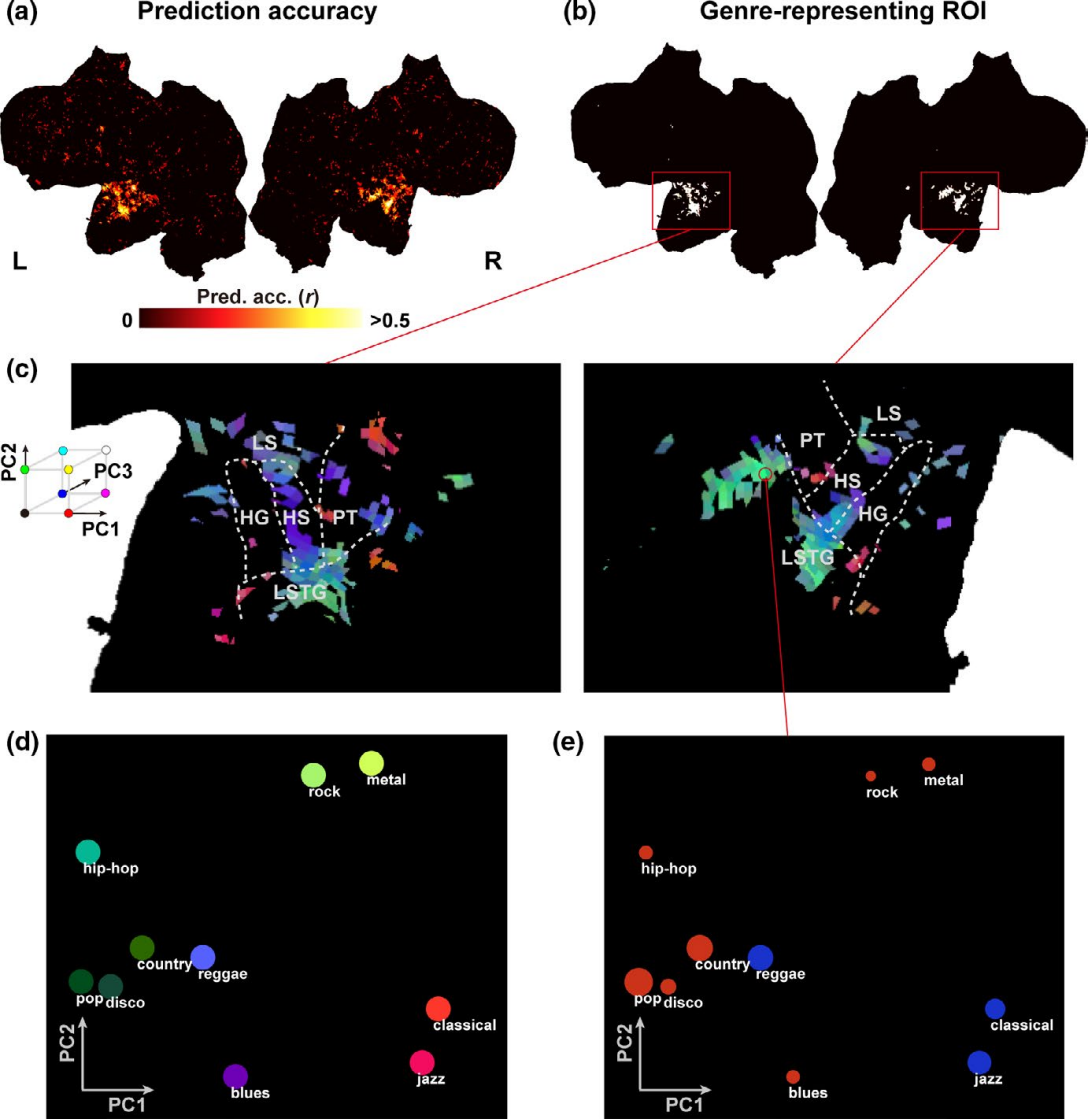
Cortical organization of music genre representations. (**A**) A cortical map of prediction accuracy using the genre‐label model (*p*<.05, FDR corrected), shown on flattened cortical sheets of participant ID01: L, left hemisphere. R, right hemisphere.**(B)**The genre‐representing ROI for participant ID01, obtained using the genre‐label model.**(C)**A cortical map of all music genres tested in the present study for participant ID01. All voxels were assigned red, green, and blue colors according to the loadings of the top three principal components (PC1‐PC3) of the genre‐label model weights (concatenated across participants). HG, Heschl’s gyrus. HS, Heschl’s sulcus. PT, planum temporale. LS, lateral sulcus. LSTG, lateral superior temporal gyrus.**(D)**Visualization of the representational relationship among the 10 music genres, mapped onto the 2‐D space based on the loadings of PC1 and PC2. The color represents the PC1‐PC3 loadings as in (**C**). The distance between each circle reflects the differences in cortical representation.**(E)**Weight values for the 10 genres extracted in a representative voxel in the right STG in participant ID01, plotted according to the same coordinates as in (**D**). The radius of each circle is equivalent to the weight value of the corresponding music genre at the target voxel (red, positive weight; blue, negative weight).

Genre‐representing cortical areas were assessed using the genre‐label model. For all participants, significant prediction accuracy was observed in the bilateral STG (*p* < .05, with FDR correction; Figure 2A, Figure S1, Table 1). To identify cortical areas that robustly represented music genres independent of sample selection, we determined the genre‐representing functional ROI for each participant (Figure 2B, Figure S2). We performed this using a resampling procedure. This analysis revealed significant prediction accuracies in the bilateral STG, and functional ROI was used as an inclusive mask in subsequent analyses.

To examine the relative contribution of each cortical voxel to the 10 music genres, we mapped the representation of the music genres on the cortical surface using PCA with genre‐label weights (Figure 2C, Figure S3). For each voxel within the ROI mask, we extracted the estimated weight matrix of the genre‐label encoding models. By averaging five temporal delays, we obtained matrices of [10 × *V*_i_] (*V*_i_: # of voxels for participant *i*, i=1,⋯,5). To obtain a general result across participants, we concatenated the weight matrix of the five participants. Then, we used PCA to perform dimensionality reduction on the aggregated weight matrix [$10\times \sum_{i=1}^{5}V_{i}$]. PCA produced a score matrix [$\sum_{i=1}^{5}V_{i}$ × 10] and loading matrix [10 × 10]. The score matrix indicates how 10 PCs are represented in each cortical voxel. The loading matrix indicates the contribution of each PC to the representation of 10 music genres. To demonstrate the representational relationship between the music genres, the 10 genres were mapped onto the 2‐D space using the loadings of PC1 and PC2 (i.e., the first and second columns of the loading matrix) as the x‐axis and the y‐axis, respectively. Genres were colored further in red, green, and blue based on the relative PCA loadings in PC1, PC2, and PC3 (i.e., the first to third columns of the loading matrix), respectively. The top 3 PC components explain 70.9% of the total variances. To represent the cortical organization of music genres for each participant, we extracted and normalized the PCA scores from each participant's voxels (i.e., for each row of the score matrix). The resultant cortical map indicates the relative contribution of each cortical voxel to the target PC. By combining the PCA scores of the top three PCs (i.e., the first to third rows of the normalized score matrix) of each participant, we visualized how each cortical voxel is represented by the 10 music genres. Each cortical voxel was colored based on the relative PCA scores of PC1, PC2, and PC3, corresponding to the color of the genre in the 2‐dimensional space. This analysis revealed various genre‐specific representations within the bilateral STG. Among the multiple subregions of STG, music genres were represented more clearly in Heschl's sulcus (HS) and the lateral STG (LSTG) than in Heschl's gyrus (HG), the planum temporale (PT), or the lateral sulcus (LS) (except participant ID03, who displayed genre‐specific activations in large brain regions including PT). Although we observed considerable individual variability, there was a marked consistency in that the contribution to pop, disco, country, and hip‐hop music (shown in green in Figure 2C, Figure S3) was larger in the LSTG than in either the HS or the HG, whereas the contribution to blues music (shown in purple) was larger around the HS.

To illustrate the relative relationships between the cortical activation patterns for different music genres, we visualized the weight values of 10 music genres in each voxel using the 2‐D coordinates derived from the top two principal components (Figure 2D). Using PCA, we embedded genre‐specific representation based on multiple cortical voxels (i.e., high‐dimensional data) into the 2‐D space, maintaining their representational similarity. These findings indicated that the activation patterns induced by classical and jazz music were relatively similar, as were those induced by rock and metal music, whereas blues and hip‐hop music seemed to have distinct activation patterns. Using the weight values of the genre‐label model, we further visualized how the 10 music genres were represented differently in each cortical voxel based on the same 2‐D coordinates (see Figure 2E for representative voxel data in participant ID01).

### 3.2 Genre‐specific representation independent from voice stimuli

**Figure 3:**
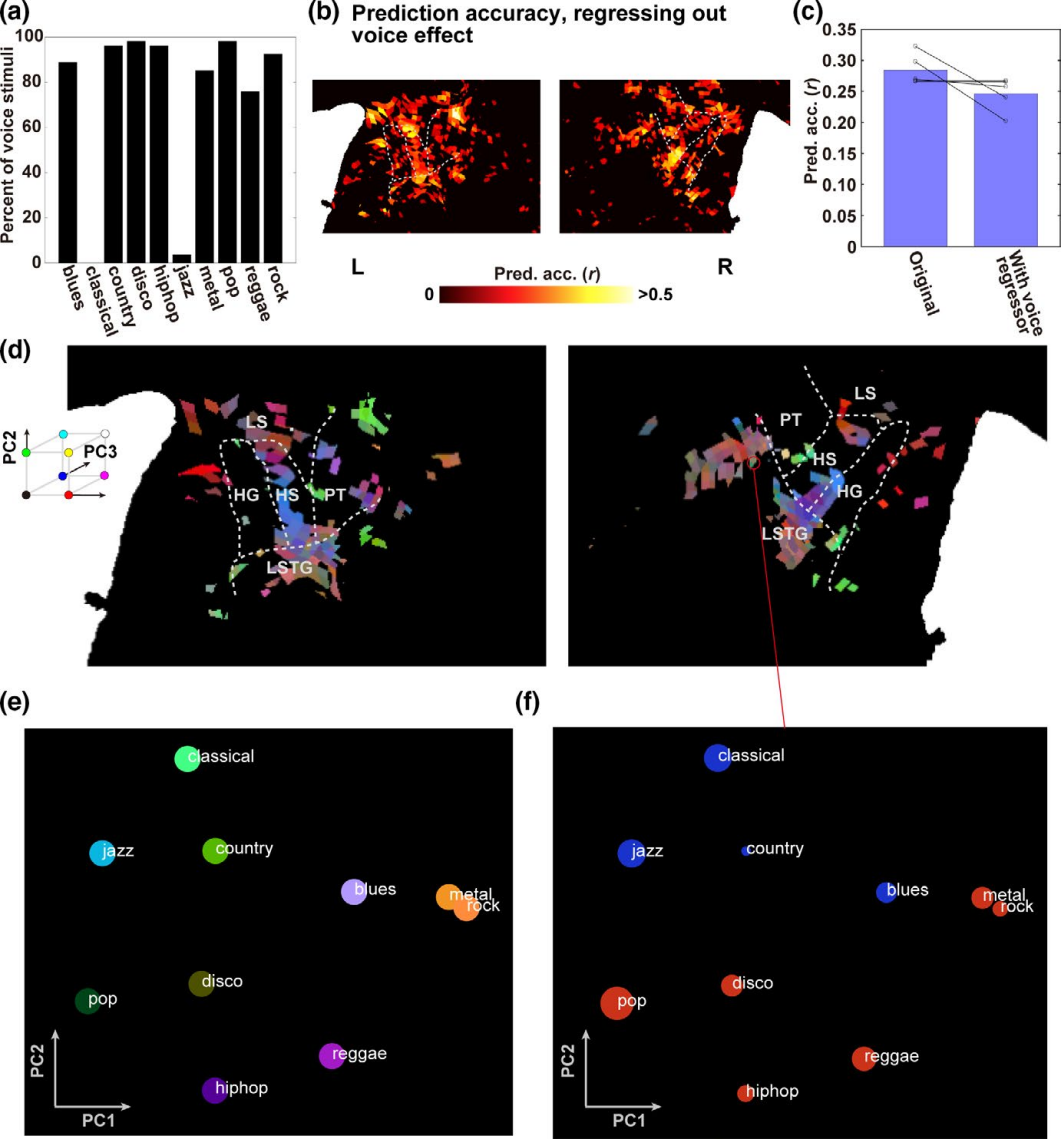
Genre‐specific representation independent from voice stimuli. (**A**) Percent of voice stimuli in each music genre. (**B**) A cortical map of prediction accuracy excluding voice model features as a regressor of noninterest, shown on the flattened cortical sheet of participant ID01. (**C**) Bar plots show average prediction accuracies across all participants, averaged within the bilateral STG ROIs, obtained using the original genre‐label model and with regressing out the voice effect. The black circles indicate each participant’s data. (**D**) A cortical map of all music genres, obtained using the genre‐label model without voice effect. All voxels were assigned red, green, and blue colors according to the loadings of the top three principal components (PC1–PC3, respectively) of the genre‐label model weights (concatenated across participants). HG, Heschl’s gyrus; HS, Heschl’s sulcus; PT, planum temporale; LS, lateral sulcus; LSTG, lateral superior temporal gyrus. (**E**) Visualization of the representational relationship among the 10 music genres, mapped onto the 2‐D space based on the loadings of PC1 and PC2. (**F**) Weight values for the 10 genres extracted in a representative voxel in the right STG.

Most of the hip‐hop and pop music clips contained voice stimuli, whereas the classical and jazz music clips did not (Figure 3A). To test whether the genre‐specific cortical representation was not explained by the inclusion of voice stimuli, we performed additional encoding model analysis. We concatenated the voice features with the original genre‐label features in the encoding model fitting. Model testing excluded the voice features from the concatenated feature matrix and the corresponding weight matrix. In this process, the voice features indicate whether certain music clip contains voice stimuli (Figure 1C). As this regressor was excluded in model testing, we regressed out the effect of voice stimuli. This model predicted activations in most of the bilateral STG ROI that were used in the original genre‐label model (77.8% ± 19.2% of voxels were significant across all participants; prediction accuracy, *r* = .246 ± .027; original genre‐label model, *r* = .284 ± .025; Figure 3B, C, Figure S4; Table 1). To examine how the cortical representation of 10 music genres is affected by regressing out the voice effect, we mapped the weight values of 10 music genres in each voxel using PCA (Figure 3D, Figure S5). We also visualized the relative relationships of 10 music genres derived from the top two PCs (Figure 3E) and their representation in each cortical voxel (Figure 3F). Similar to the results in Figure 2, the activation patterns induced by classical and jazz music were relatively similar, as were those induced by rock and metal music, whereas blues and hip‐hop music had distinct activation patterns. These findings suggest that the genre‐specific activation patterns were not explained merely by the inclusion of the voice stimuli.

### 3.3 Genre‐specific brain activity was explained by the spectro‐temporal modulation of music genres

**Figure 4:**
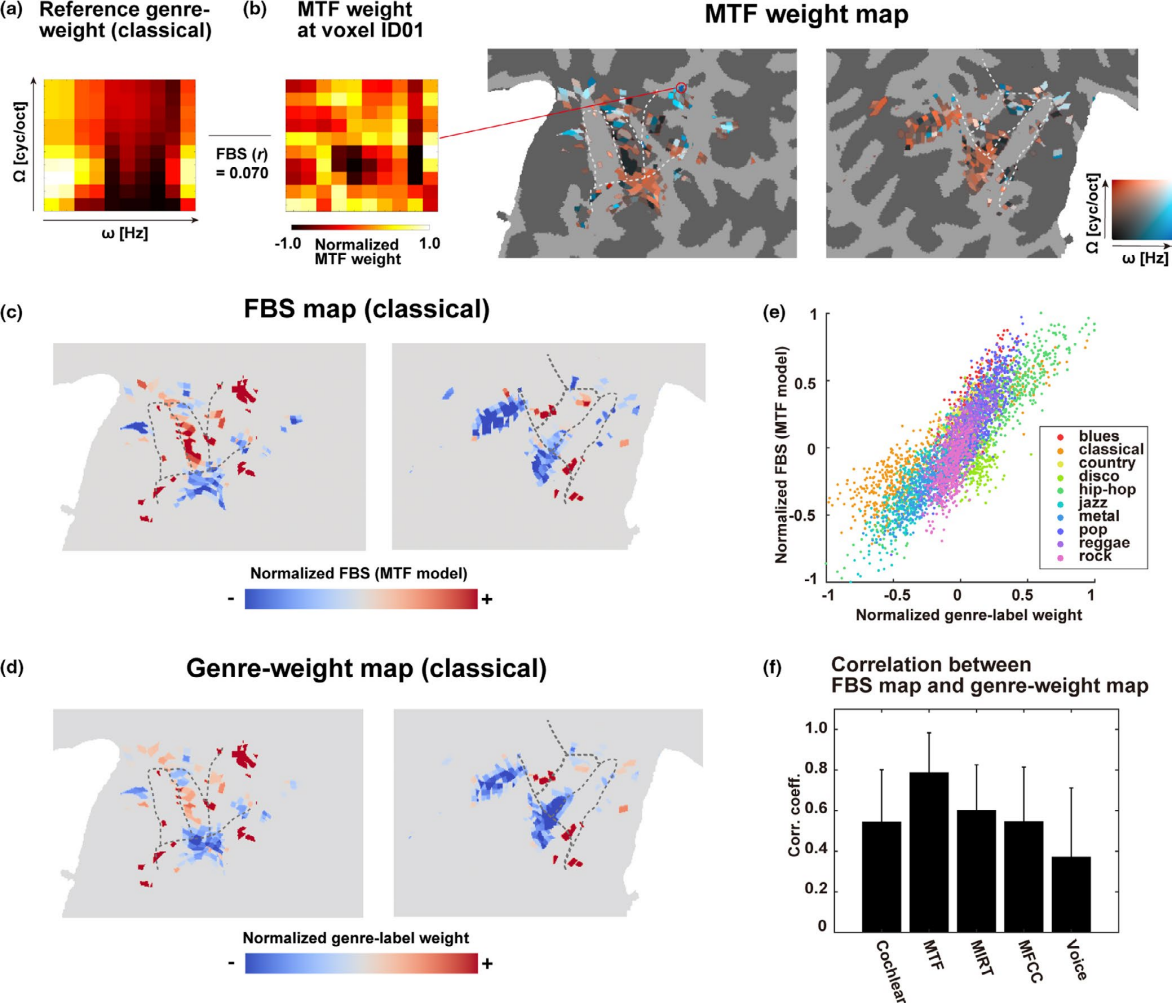
Contribution of spectro‐temporal features to music genre representation.**(A)**Examples of the averaged modulation profiles for classical music. Modulation‐transfer function (MTF) model weights were projected on a 2‐D plot of the spectral modulation Ω (cyc/oct) and temporal modulation rates ω (Hz).**(B)**Spectro‐temporal modulation of the cortical voxels determined in participant ID01. Weight vectors of the MTF model were averaged for 20 central frequencies. Each cortical voxel was assigned the maximum spectral/temporal modulation rate of that voxel. The averaged modulation profile of an example voxel is shown on the left panel. Feature‐brain similarity (FBS) was calculated as a Pearson’s correlation coefficient between the modulation profile in each cortical voxel and the reference modulation profile of each music genre.**(C)**FBS cortical maps of participant ID01 obtained from the spectro‐temporal modulations of classical music (i.e., MTF model features). Data were normalized and projected on the inflated cortical map (red, positive weight; blue, negative weight).**(D)**The normalized weights of the genre‐label model were projected on the inflated cortical map (genre‐weight map; red, positive weight; blue, negative weight), for classical music.**(E)**The 2‐D scatterplot of voxel values of the normalized genre‐weight map and the FBS map of the MTF model (**C**and**D**) taken from all voxels in the genre‐representing ROI of participant ID01, and overlaid with 10 music genres.**(F)**Pearson’s correlation coefficients between voxels in the FBS map and those in the genre‐weight map for participant ID01 for the cochlear, MTF, music information retrieval toolbox (MIRT), and mel‐frequency cepstral coefficient (MFCC), voice; see Methods), averaged for 10 music genres. The MTF model provided the highest similarity (Wilcoxon signed‐rank test,*p*<.010 for all the models).

Next, we tested how well acoustic feature‐based genre specificity corresponds to brain‐based feature specificity. To achieve this, we extracted the acoustic features of each music stimulus using the five acoustic models (Cochlear, MTF, MIRT, MFCC, and voice). In this study (Figure 4A‐E), we only show the MTF model results for the purpose of visualization; however, the following analyses were performed similarly for all acoustic models. Spectro‐temporal modulation of each music genre was evaluated according to the feature matrix used for encoding model fitting. Interpretable spectro‐temporal information was obtained for each MTF feature by restoring the MTF feature matrix to the original size through multiplying it by the transposed PCA coefficient matrix. Moreover, we transformed the MTF weight matrix by multiplying it by the PCA loading matrix. In order to visualize the MTF model, we further averaged the feature values obtained at the 20 central frequencies for each of the 10 × 10 combinations of spectral modulation Ω (cyc/oct) and temporal modulation ω (Hz). Genre‐specific feature vectors were calculated for the other models using the same procedure. By averaging the MTF features for the 48 clips of the same music genre in the training dataset, we obtained reference MTF features for the 10 music genres (Figure 4A). We then obtained the MTF features of each cortical voxel using the weight matrix of the MTF model (Figure 4B; see Figure S6 for a weight map of the other models). The response of each cortical voxel to spectro‐temporal modulation was assessed using the transformed weight matrix. Voxel‐specific weight vectors were calculated by averaging the MTF weight values of the 48 training clips for each genre. Voxel‐specific weight vectors were calculated for the other models using the same procedure. In order to visualize the MTF model, we further averaged the weight values obtained at the 20 central frequencies for each of the 10 × 10 combinations of spectral modulation Ω (cyc/oct) and temporal modulation ω (Hz).

To examine whether genre‐representing activation patterns were explained by the extracted features, we calculated the Pearson's correlation coefficients between the reference genre‐specific feature vector and the voxel‐specific weight vector in each cortical voxel. Through this analysis, we obtained an FBS cortical map of each music genre based on its MTF features (Figure 4C, Figure S7). The FBS map based on the MTF model was very similar to the cortical map obtained for genre‐label weight (genre‐weight map, Figure 4D, Figure S8), and they were significantly correlated for all music genres (Pearson's correlation coefficient, *p* < .001, with Bonferroni correction; Figure 4E, Figure S9). A significant correlation was observed consistently across all participants (*r* = .729 ± .111), indicating that the different spectro‐temporal modulations of the 10 music genres explained genre‐specific activity patterns. The FBS map was also obtained for the other acoustic models, and we found that the MTF model outperformed the other models (cochlear, MIRT, MFCC, and voice models) in terms of the mean correlation between the FBS maps and the genre‐weight maps (Wilcoxon signed‐rank test, *p* < .020 for participants except for ID02; Figure 4F, Figure S10). Although we used earphones that attenuate scanning noise, the remaining noise could have degraded the stimulus quality and modulated activity patterns in the auditory cortex (Peelle, 2014). To evaluate the effects of scanning noise on our results, we therefore performed additional analyses (Figure S11). Specifically, we recorded the MRI noise inside the scanner using an MRI‐compatible microphone and added this noise to the original auditory stimuli. Since the relative intensity of noise depends on the depth of insertion, we added the noise with three different relative intensities (0.2, 0.5, and 1.0 to the mean RMS of the original stimuli). For participants ID01, ID04, and ID05, we found that the MTF model exhibited the largest correlation coefficients between the FBS maps and genre‐weight maps independent of relative noise intensities (Wilcoxon signed‐rank test, *p* < .050; except for the comparison between the MTF and cochlear models for participant ID01 with noise intensities of 1.0 and 0.5, for which *p* = .065, and between the MTF and MIRT models for participant ID04 with a noise intensity of 0.5, for which *p* = .084). For participants ID02 and ID03, a relative noise intensity of 0.2 more clearly demonstrated the advantage of the MTF model (Wilcoxon signed‐rank test, *p* < .010). These results suggest that the MTF model accurately captured genre‐specific cortical organization in the bilateral STG.

### 3.4 Genre classification accuracy based on brain activity, behavior, and acoustic models

**Figure 5:**
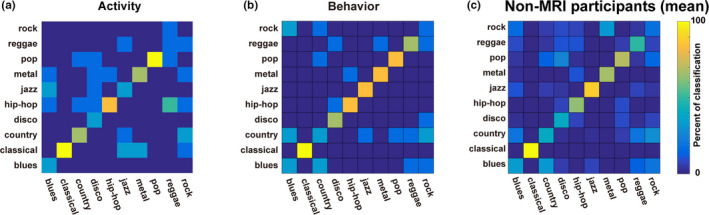
The modulation‐transfer function (MTF) model explains the genre representational specificities of brain activity and behavior.**(A)**The confusion matrix based on the brain activity of participant ID01 using the decoding model approach. For each column of correct music genres, the percentage of classified music clips was plotted on the row of classified music genres.**(B)**The confusion matrix based on the behavioral data of participant ID01.**(C**) The mean confusion matrix based on the behavioral data of the non‐MRI participants. For each column of music genres, the percentage of correctly classified music clips was plotted on the row of the classified music genres. Error bar, SD.

In the MRI experiments in this study, participants listened passively to music stimuli and did not carry out any genre classification tasks during scanning. To confirm that participants’ brain activity captured sufficient information to distinguish different music genres in the current experimental setting, we conducted a genre classification based on brain activity using a decoding model approach (see Methods). We evaluated the confusion matrix, along with the classification accuracy (the diagonal elements of the confusion matrix), using cortical activation within the genre‐representing ROI masks (Figure 5A, Figure S12). The results of classification varied across genres, in that classical music was always classified accurately (average classification accuracy, 100%), whereas classification performance for rock music was poor across all participants (13.3% ± 18.3%). We also found that participants tended to classify reggae music as hip‐hop music (confusion from reggae to hip‐hop, 43.3% ± 14.9%), whereas they tended to classify rock music as country music (confusion from rock to country, 33.3% ± 11.8%). The activity‐based confusion matrices were highly consistent across all participants (Spearman's correlation coefficient, *ρ = *0.553 ± 0.062; *p* < .001 for all combinations of participants).

To investigate how brain activation associated with music genre is related to behavioral performance during genre classification, we conducted an additional behavioral test for each participant (MRI participants) after the MRI scanning (Figure 5B, Figure S13). The confusion matrix revealed that participants’ genre classification performance varied for each genre, in that classical music was always accurately recognized (average classification accuracy, 100%), while rock music was less accurately recognized across all participants (36.7% ± 13.9%). Behavioral confusion matrices identified brain activity‐like error tendencies, such that rock music tended to be classified as country music (confusion from rock to country, 20.0% ± 13.9%). The behavior‐based confusion matrices were highly consistent across all participants (Spearman's correlation coefficient, *ρ = *.669 ± 0.038; *p* < .001 for all combinations of participants). The confusion matrices of behavior and brain activity were significantly correlated for all participants (Spearman's correlation coefficient, *ρ = *.438 ± 0.081, *p* < .001), suggesting that the genre representational specificity of human behavior mimicked that of brain activity.

Further, because the MRI participants listened to the music stimuli twice (once in the MRI scanner and again in the behavioral test), there may have been a learning effect. Moreover, it is necessary to test whether the five MRI participants have similar perceptual properties for music genres as those of the general population. To confirm the generalizability of the behavioral results of these participants, we recruited an additional 21 participants for the behavioral tests only (non‐MRI participants, Figure 5C). The non‐MRI participants exhibited variable genre classification accuracy (mean ± *SD*, 56.3% ± 6.1%; max, 68.3%; min, 43.3%), with performances similar to those of the MRI participants, in that they always recognized classical music accurately (100%) whereas they did not always do so with rock music (17.5%). Accordingly, the average behavioral confusion matrices of the MRI participants and the non‐MRI participants were significantly correlated (*ρ* = .826, *p* < .001).

## 4 DISCUSSION

Using fMRI, the current study revealed the cortical organization underlying different music genres. As the genre‐label model did not assume any acoustic properties, we used genre‐weight maps (Figure 4D) to reflect music genre information in general. Thus, it was important to obtain similar weight patterns between the genre‐label model (Figure 4D) and the FBS map based on the MTF model (Figure 4C). The FBS map shows how the spectro‐temporal modulation of each cortical voxel corresponds to the reference spectro‐temporal modulation profile for each music genre. Thus, it is likely that the weight values in the bilateral STG for the genre‐label model were determined by the degree to which each STG voxel’s spectro‐temporal modulation property resembles that of the music stimuli.

Among the multiple subregions in STG, music genres were represented more clearly in both HS and LSTG than in the other subregions. Previous studies on frequency‐selective (i.e., tonotopic) maps of the human STG have indicated that the primary auditory cortex (A1) is located around the posterior part of HG and HS accompanied by a gradient of low‐ to high‐frequency selectivity from the anterior to posterior directions (Ahveninen et al., 2016; Humphries et al., 2010; Leaver & Rauschecker, 2016; Moerel et al., 2014). While cochlear features correspond to positions in the frequency axis and may therefore reflect tonotopic properties (see Figure 1C), the MTF model further captures the modulation property around each position on the frequency axis. Santoro et al. (2014) showed that the MTF model outperformed the cochlear model in terms of predicting STG activation in response to natural sound stimuli (Santoro et al., 2014), which is consistent with the current results. LSTG has been reported to represent different sound categories such as the sound of a guitar versus. voice of a cat (Staeren et al., 2009), and it exhibits human speech‐selective activation (Leaver & Rauschecker, 2010; Norman‐Haignere et al., 2015). The MTF model captures detailed spectro‐temporal modulation properties both in human speech (Elliott & Theunissen, 2009) and in musical instruments (Patil et al., 2012), which may explain the more general acoustic features that can encompass the feature space of simple categorical models such as genre‐label or voice models. To summarize, the spectro‐temporal modulations obtained in our study seem to reflect the general processing properties of auditory stimuli in the bilateral STG.

Several studies have reported that perceived music genres can be decoded from brain activity. Ghaemmaghami and Sebe (2017) used magnetoencephalogram and electroencephalogram datasets to classify musical stimuli as either pop or rock using SVM (Ghaemmaghami & Sebe, 2016). Further, Case y (2017) and Sengupta et al. (2018) used fMRI data with five distinct music genres, followed by activity‐based multi‐class classification using SVM. However, these studies did not provide answers to how cortical representations of music genres contribute to genre classification. Collectively, the present findings demonstrate the underlying mechanisms of such activity‐based genre classification.

We investigated classification accuracy using five models (cochlear, MTF, MIRT, MFCC, and voice). Both the MFCC and the MIRT models were developed in the field of computational science and have been employed previously in studies of music‐induced brain activity (Alluri et al., 2012; Güçlü et al., 2016; Toiviainen et al., 2014). The cochlear model has been employed to test cortical activation in the spectral domain (de Heer et al., 2017); however, it cannot capture the dynamic temporal modulation of spectra (see Figure 1C). The MTF model was constructed based on the physiological properties of neurons in the auditory cortex (Chi et al., 2005) and is used widely in neuroscience research into auditory perception (Norman‐Haignere et al., 2015; Patil et al., 2012; Santoro et al., 2014, 2017). Therefore, it is likely that the MTF model is more biologically plausible for addressing the auditory processing of music genres. Our current findings are consistent with this view, because, of all the models, the MTF model showed the highest correlation coefficients between the FBS maps and genre‐weight maps (Figure 4F).

One might argue that the fMRI signal change is too slow to capture the rapid acoustic features of music stimuli and that this could affect the model performance with up‐tempo (e.g., metal) and slow‐tempo (e.g., classical) music genres. However, the MTF model includes temporal modulations of frequency (from 2.8 to 64.0 Hz) and the estimated model weights show signals in high temporal modulation rates (e.g., Figure 4B), suggesting that this model can capture the fine‐scale musical information necessary to distinguish relatively up‐tempo music genres (e.g., metal and hip‐hop). Indeed, the difference in decoding accuracy (in Figure 5A) is not explained by the difference in tempo, given that both classical and hip‐hop music showed higher decoding accuracies.

In the MRI experiments in this study, participants listened passively to music stimuli and did not carry out any genre classification tasks during scanning. It could be argued that we did not confirm that the participants listened attentively to the stimuli and that we overlooked the brain regions activated for top‐down decision‐making on music genre classification. To address this, we conducted behavioral experiments of genre classification for MRI participants (Figure 5B) and confirmed that there were significant correlations between the confusion matrices based on brain activation and behavior. These findings suggested that passive listening to music stimuli captured enough brain information for use in behavioral music genre classification.

In this study, we adopted a small‐N design (five participants). The small number of subjects is compensated for by the large number of samples for each participant (i.e., three hours). The small‐N design has attracted substantial attention in recent studies combining fMRI data and machine learning (Smith & Little, 2018). Instead, of group‐level statistical analyses, as are often used in conventional neuroimaging, we performed subject‐wise analyses. The correspondence of genre representation among participants was confirmed using Pearson's correlation of confusion matrices for both activity‐based decoding and behavior‐based analyses. In contrast, cortical organization differed across participants. For instance, the left HS showed much larger *SD* of prediction accuracy than the other anatomical ROI (genre‐label model: left HS, *SD* = 0.085; mean *SD* across other ROIs = 0.026; Table 1), indicating that the left HS is the most sensitive region to the individual variability of music genre representation.

It is worth considering whether linguistic factors could explain genre‐specific organization because most classical and jazz pieces employed in the current study were instrumental (i.e., without human voice), whereas other genres included the human voice (Figure 3A). Previous studies have reported voice‐selective and nonvoice‐selective cortical areas around STG (Kell et al., 2018; Leaver & Rauschecker, 2010; Norman‐Haignere et al., 2015). However, our study demonstrated that such linguistic factors do not explain genre‐specific patterns. The genre‐label model in our study predicted brain activity even after regressing out voice‐related features (Figure 3B, C). In addition, we also showed that classical and jazz music were not confused with each other (see the confusion matrices in Figure 5) and that some music genres containing voice stimuli were not confused with each other (e.g., hip‐hop and country music). Thus, it is likely that genre‐specific activation patterns in the bilateral STG reflected detailed spectro‐temporal modulation even within nonvoice music pieces.

Although we have shown that FBS maps and genre‐weight maps largely corresponded, the correspondence is not perfect. There are several possible reasons for such imperfection. First, the FBS maps and genre‐weight maps were susceptible to the noise of brain activity. The upper limit of prediction accuracy also affects the accuracy of both FBS maps and genre‐weight maps. Second, the MTF might not be the best model. Indeed, an acoustic model might exist that captures more detailed characteristics of music genres. Third, some nonacoustic features (such as the participants’ preference, knowledge, and experience related to music) may play important roles in producing the genre‐specific cortical organization. Further research is therefore required to clarify the detailed neural basis of music categorization.

## 5 CONCLUSION

In conclusion, music genre categories are represented in the bilateral STG in a genre‐specific way and that spectro‐temporal modulation profiles extracted from the music pieces themselves can be used to model these representations. To summarize, our finding suggest that it may be possible to model the categorization of complex auditory stimuli based on brain activity.

## 6 ETHICS APPROVAL

This experiment was approved by the ethics and safety committee of the National Institute of Information and Communications Technology in Osaka, Japan.

## CONFLICT OF INTEREST

The authors declare no competing financial interests.

## **AUTHOR** **CONTRIBUTION**

T.N., N.K., and S.N. designed the study and wrote the manuscript. T.N. and N.K. carried out the experiment. T.N. analyzed the data with support from N.K.

### Peer Review

The peer review history for this article is available at https://publons.com/publon/10.1002/brb3.1936.

## Supporting information

Supplementary Material

## Data Availability

The data that support the findings of this study are available from the corresponding author upon request.
