# Supplementary material for: Correspondence of categorical and feature‐based representations of music in the human brain
Source: Brain Behav. 2020 Nov 8;11(1):e01936. doi: 10.1002/brb3.1936 (PMC7821620; doi:10.1002/brb3.1936)
Supplement: Supplementary file 1 — Supplementary Material [file BRB3-11-e01936-s001.pdf]

# Correspondence of categorical and feature-based representations of music in the human brain

Tomoya Nakai, Naoko Koide-Majima, and Shinji Nishimoto

Supporting information

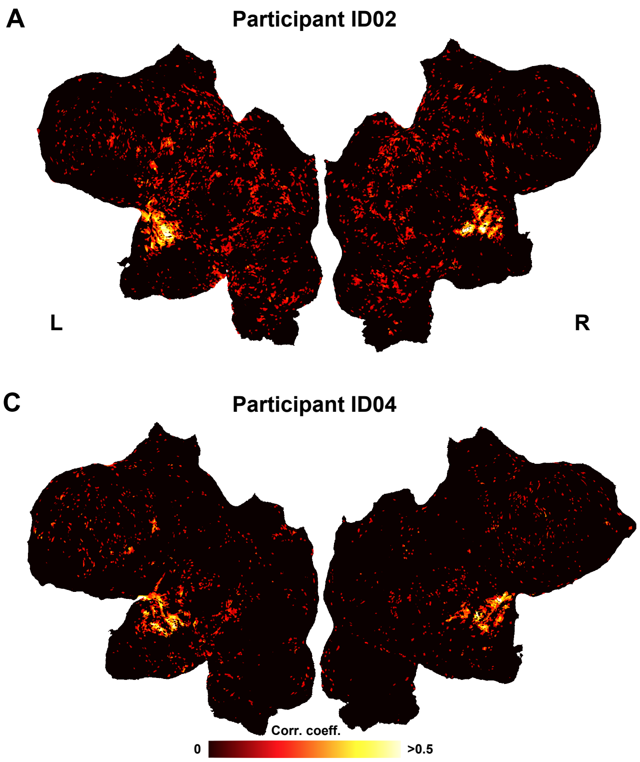

Figure S1. Prediction accuracy of the genre-label model. Cortical maps of prediction accuracy using the genre-label model ($p < 0.05$, FDR corrected) shown on flattened cortical sheets for participants ID02-05.

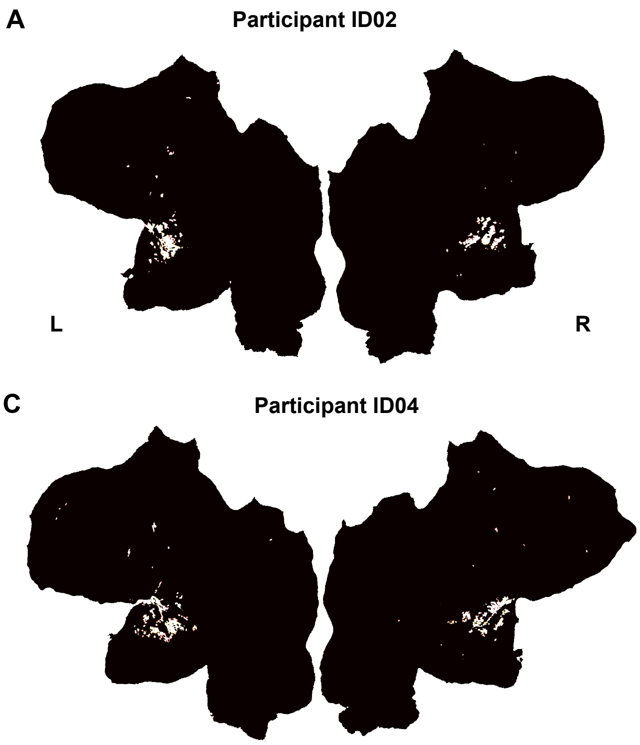

Figure S2. Genre-representing ROIs. Genre-representing ROIs for participants ID02-05 shown on flattened cortical sheets.

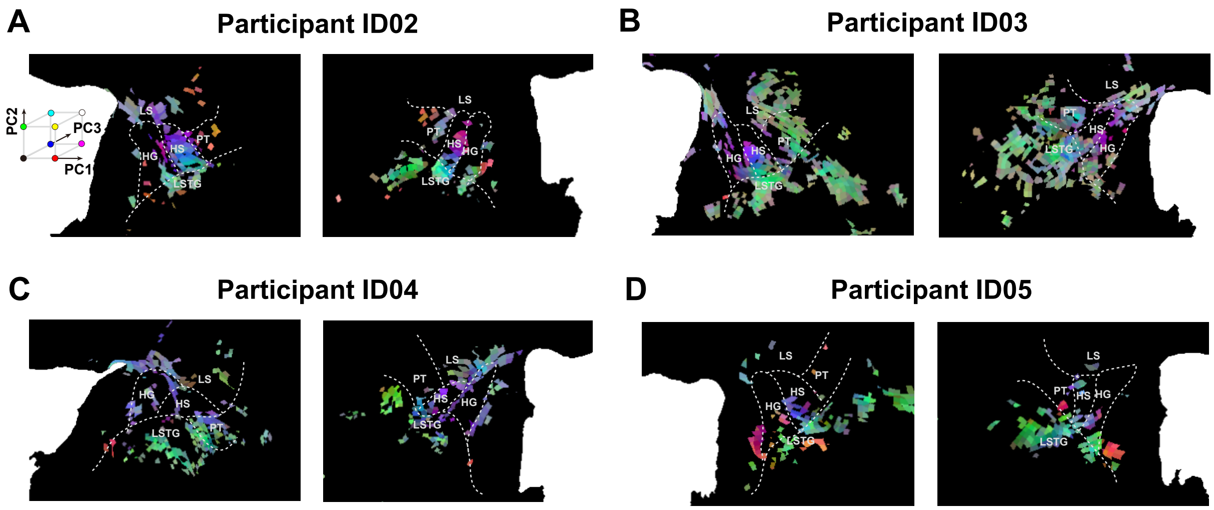

Figure S3. Cortical organization of music genres. Cortical maps of all genres tested in the present study for participants ID02-05. All voxels were assigned red, green, and blue colors according to the loadings of the top three principal components (PC1-PC3) and the genre-label model weights (concatenated across participants). HG, Heschl's gyrus. HS, Heschl's sulcus. PT, planum temporale. LS, lateral sulcus. LSTG, lateral superior temporal gyrus.

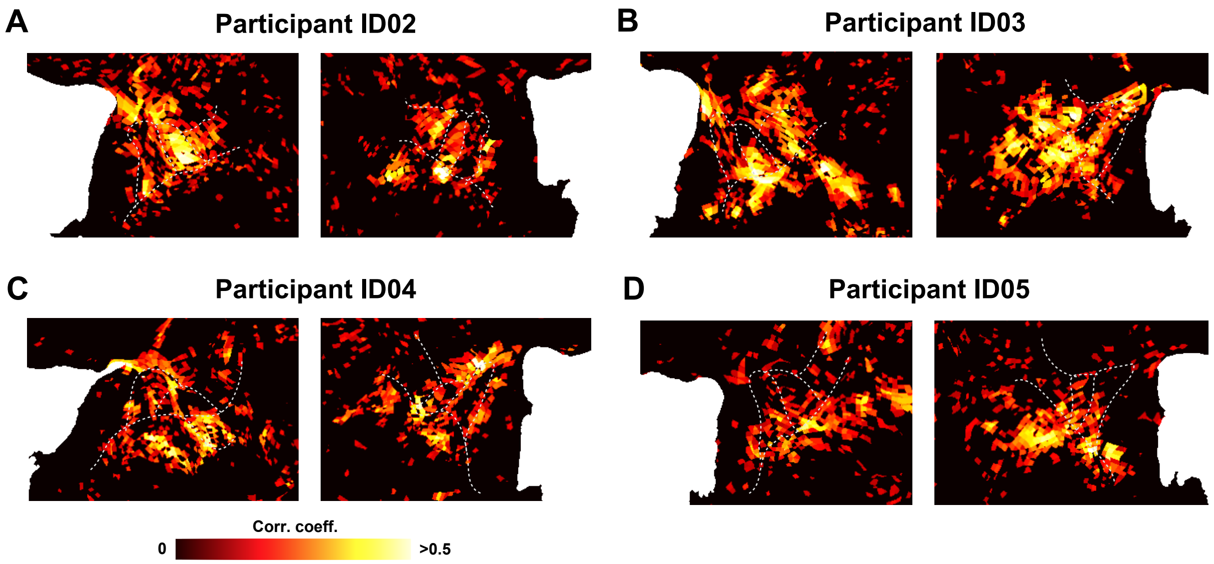

Figure S4. Prediction accuracy of the genre-label model excluding the voice effect. Cortical maps of prediction accuracy using the genre-label model, excluding the voice effect ($p < 0.05$, FDR corrected), shown on flattened cortical sheets for participants ID02–05.

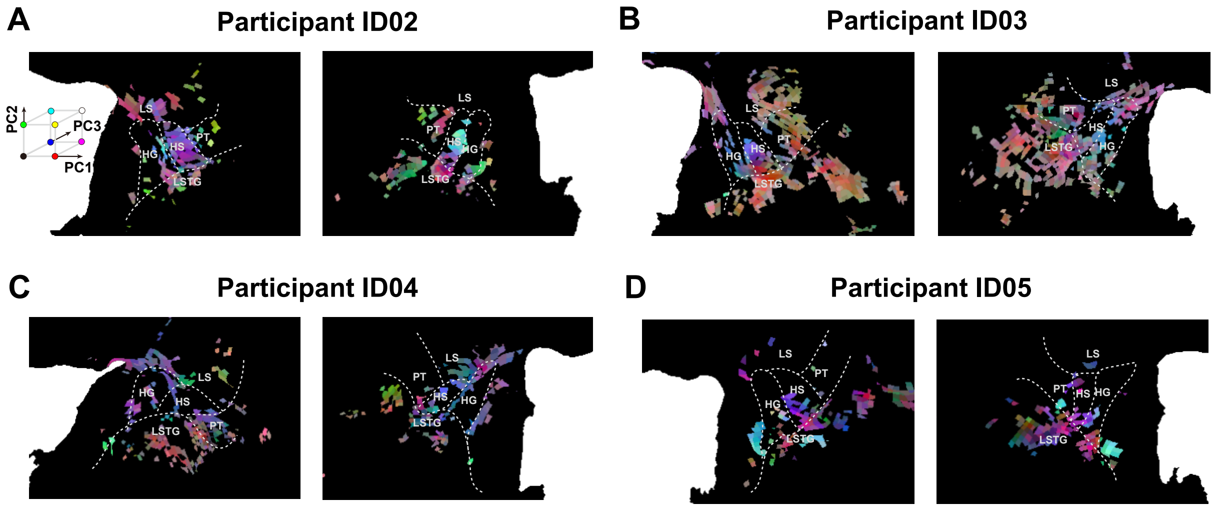

Figure S5. Cortical organization of music genres excluding the voice effect. Cortical maps of all genres tested in the present study for participants ID02–05, excluding the voice effect. All voxels were assigned red, green, and blue colors according to the loadings of the top three principal components (PC1–PC3, respectively) and the genre-label model weights (concatenated across participants). HG, Heschl's gyrus; HS, Heschl's sulcus; PT, planum temporale; LS, lateral sulcus; LSTG, lateral superior temporal gyrus.

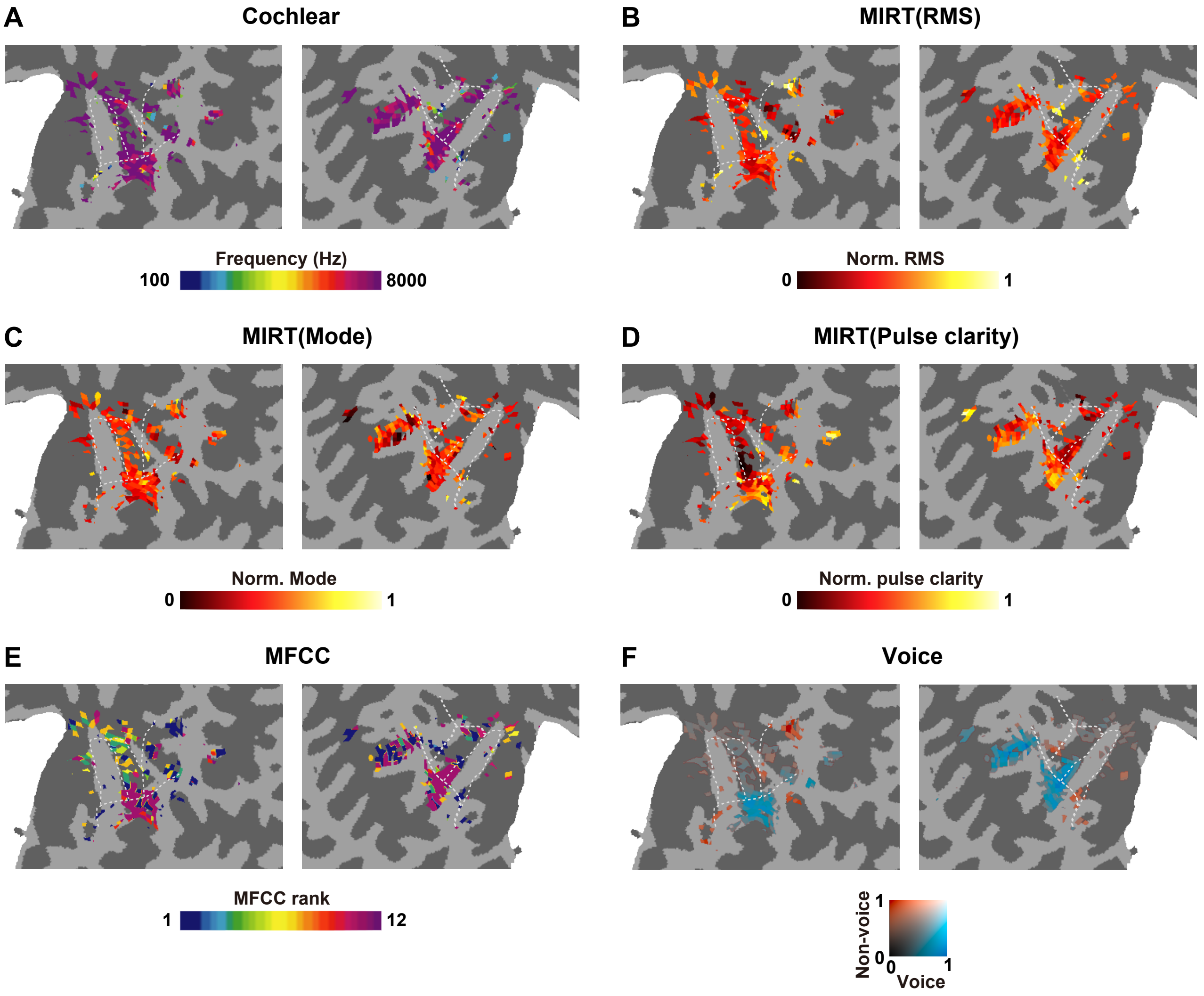

Figure S6. Weight map of other models. Cortical maps of weight values are shown for participant ID01 for the cochlear, modulation-transfer function (MTF), music information retrieval toolbox (MIRT), mel-frequency cepstral coefficient (MFCC), and voice models. For the MIRT model, we selected three features (root mean square (RMS), mode, and pulse clarity) as representatives of sound intensity, harmony, and rhythm (respectively).

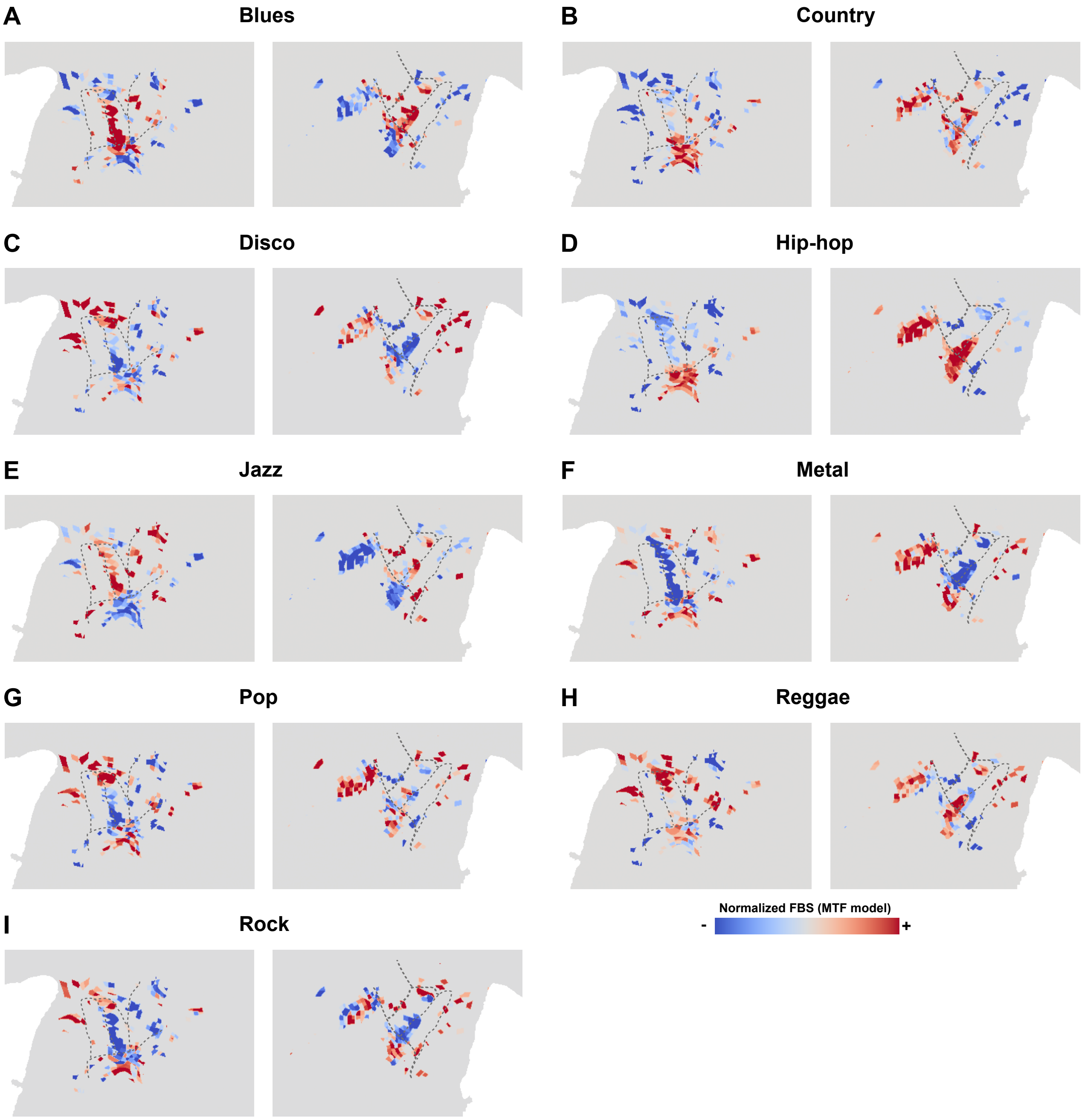

Figure S7. Feature-brain similarity maps. Feature–brain similarity maps are shown for participant ID01, obtained from the spectro–temporal modulations of blues, country, disco, hip-hop, jazz, metal, pop, reggae, and rock music. Data were normalized and projected onto the inflated cortical map (red, positive weight; blue, negative weight).

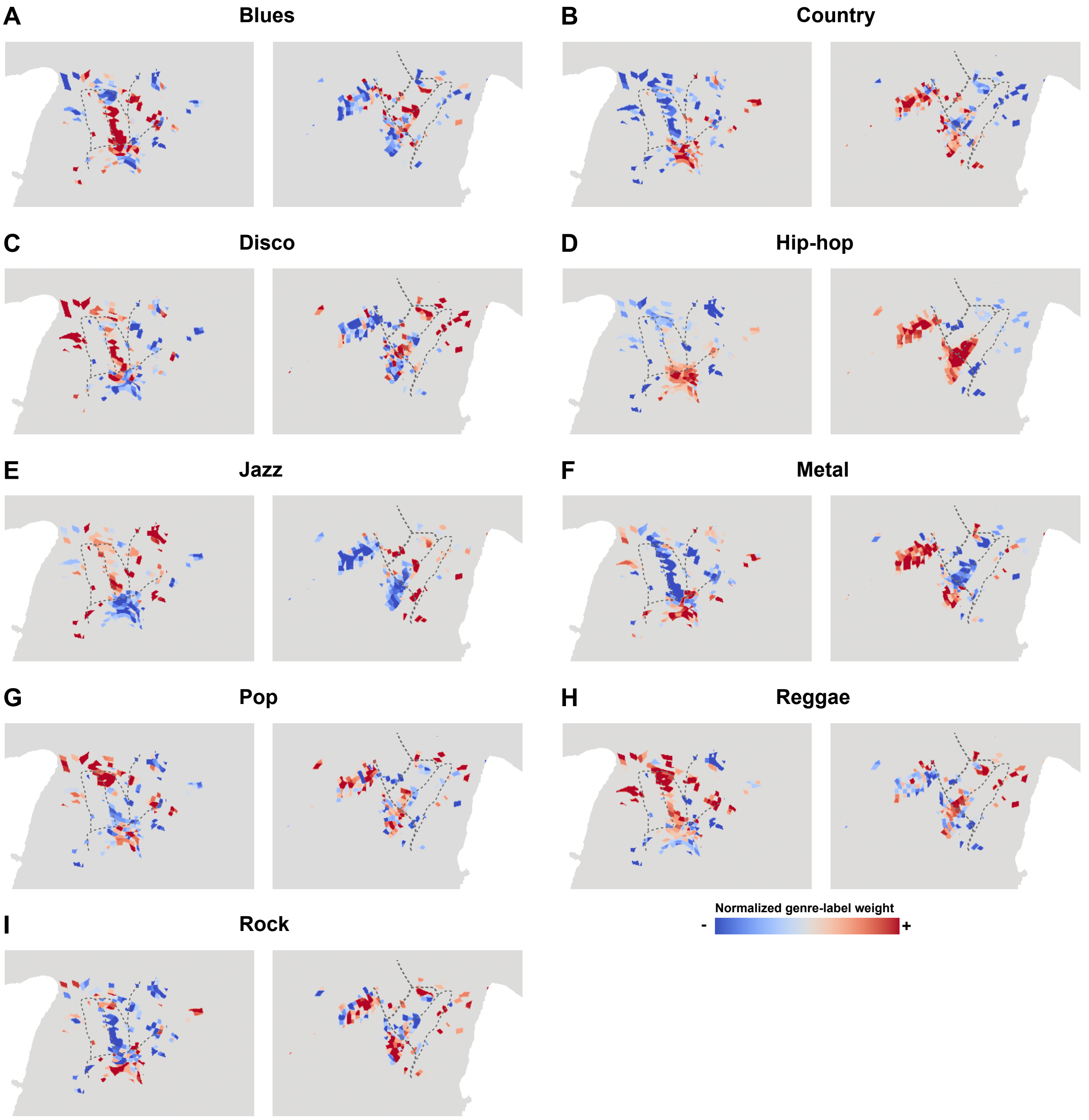

Figure S8. Genre-weight maps. The normalized weights of the genre-label model were projected onto the inflated cortical map of participant ID01 (genre-weight map: red, positive weight; blue, negative weight) for blues, country, disco, hip-hop, jazz, metal, pop, reggae, and rock music.

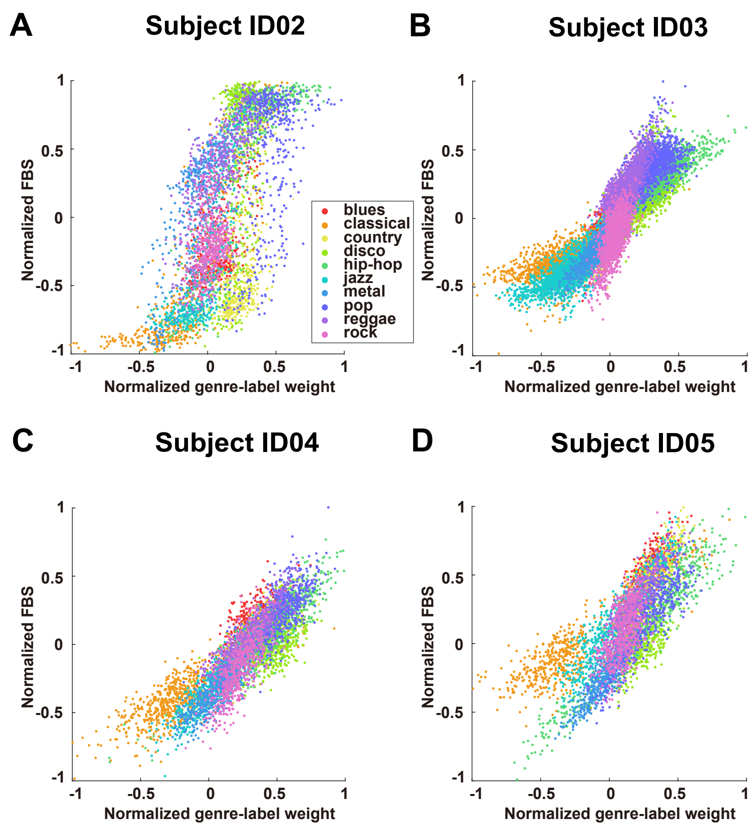

Figure S9. Scatter plots of genre-weight and feature-brain similarity values. 2-D scatterplots of voxel values of normalized genre-weight map and feature-brain similarity (FBS) maps of the modulation-transfer function (MTF) model taken from all voxels in the genre-representing ROI of subjects ID02-05 and overlaid with 10 music genres.

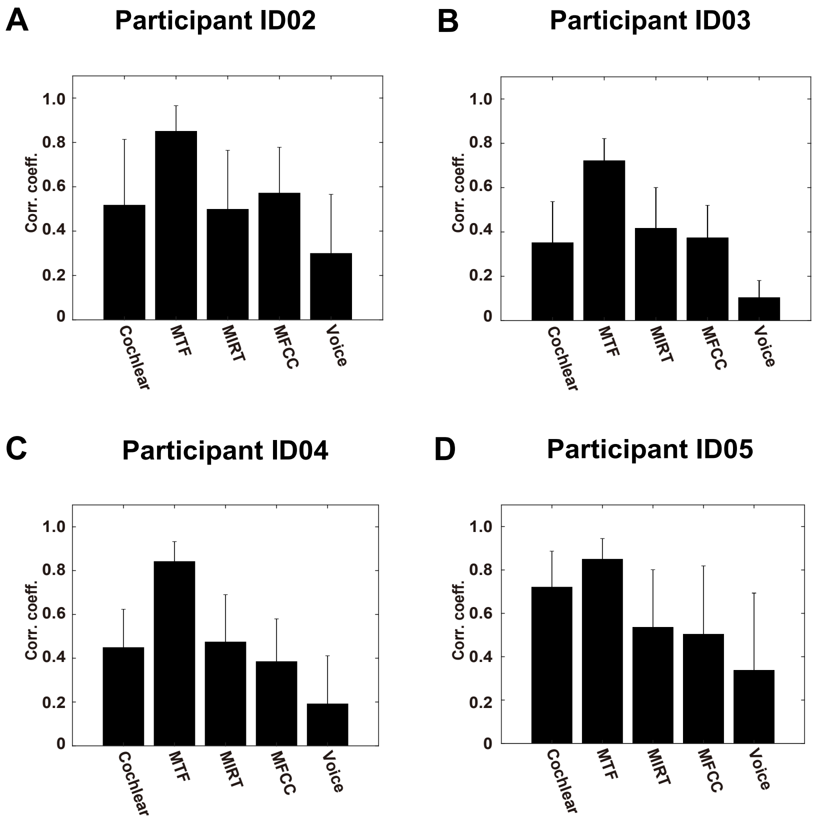

Figure S10. Correlation between genre-weight maps and feature-brain similarity maps. Pearson's correlation coefficients between voxels in the genre-weight map and those in the feature-brain similarity (FBS) map for participants ID02-05, for the Cochlear, modulation-transfer function (MTF), music information retrieval toolbox (MIRT), mel-frequency cepstral coefficient (MFCC), and voice features, averaged for 10 music genres. Error bar, SD.

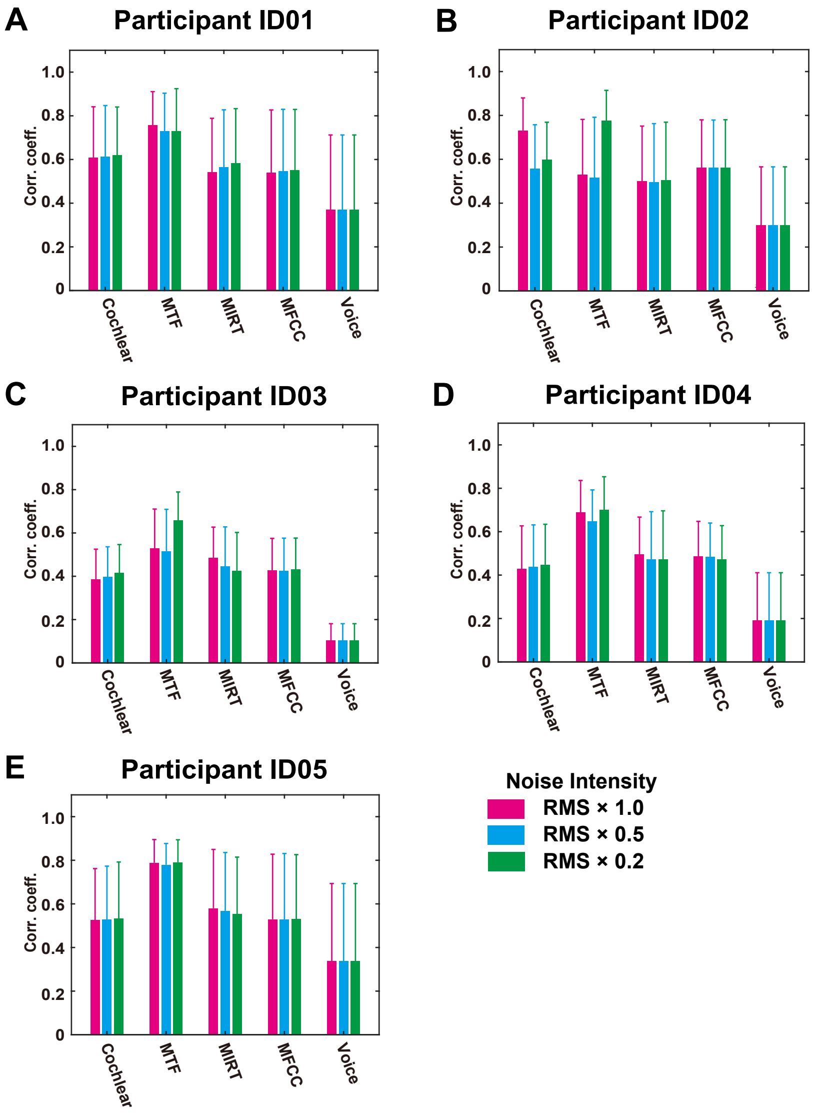

Figure S11. Correlation between genre-weight feature-brain similarity maps with scanning noise. Pearson's correlation coefficients between voxels in the genre-weight map and those in the feature-brain similarity map for participants ID01-05, shown for the cochlear, modulation-transfer function (MTF), music information retrieval toolbox (MIRT), mel-frequency cepstral coefficient (MFCC), and voice features, averaged for 10 music genres. The cochlear, MTF, MIRT, and MFCC models were constructed based on the noise-added auditory stimuli. Scanning noise was added with three different intensities (1.0, 0.5, and 0.2 to the mean root mean square of the original stimuli, as indicated in purple, blue, and green, respectively). Error bars: SD.

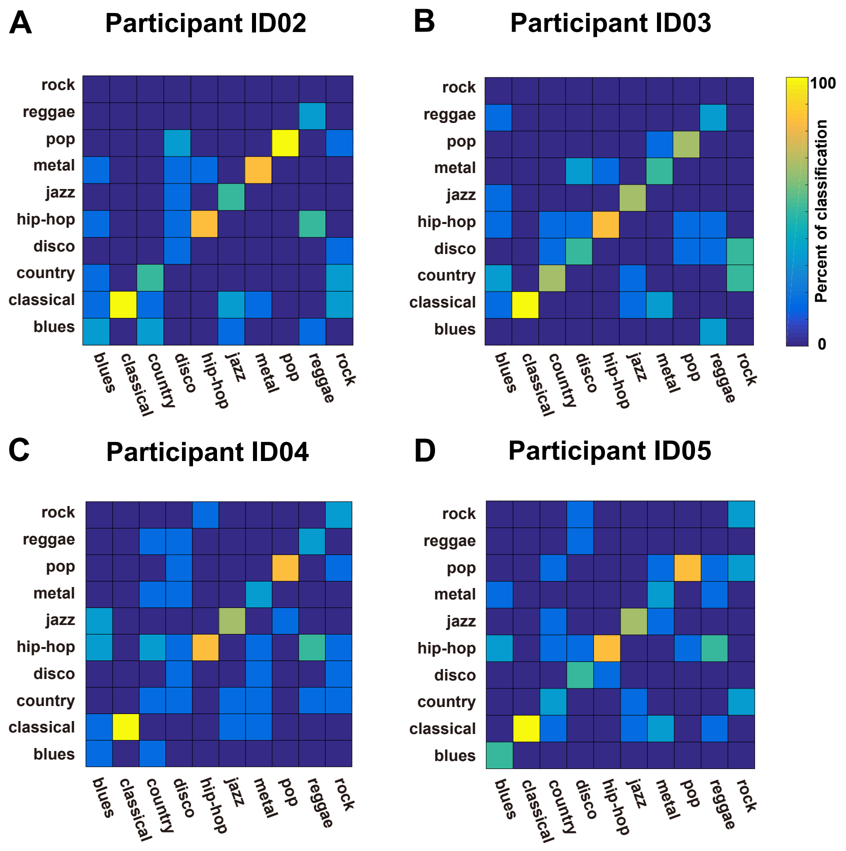

Figure S12. Activity-based genre classification. Confusion matrix based on the brain activity of participants ID02-05. For each column of the correct music genres, the percentage of classified music clips were plotted on the row of the classified music genres.

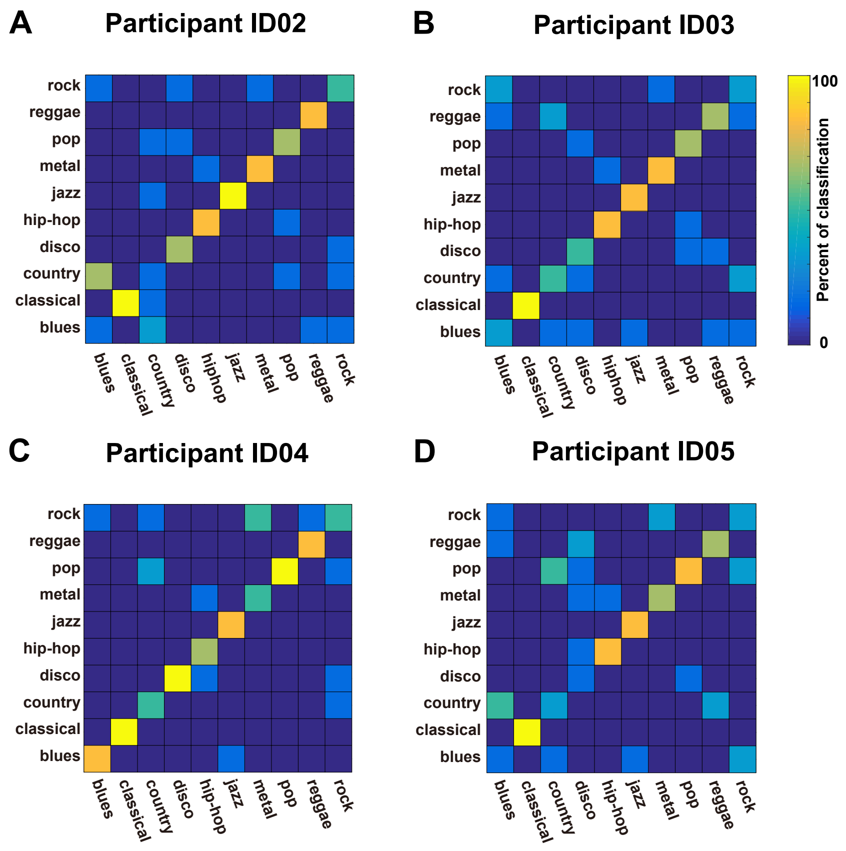

Figure S13. Behavior-based genre classification. Confusion matrix based on the behavioral data of participants ID02-05.
